# Infections and Ischemic Stroke Outcome

**DOI:** 10.1155/2011/691348

**Published:** 2011-06-28

**Authors:** Katarzyna Grabska, Grażyna Gromadzka, Anna Członkowska

**Affiliations:** ^1^2nd Department of Neurology, Institute of Psychiatry and Neurology, 02-957 Warsaw, Poland; ^2^Department of Clinical and Experimental Pharmacology, Medical University of Warsaw, 00-927 Warsaw, Poland

## Abstract

*Background*. Infections increase the risk of ischemic stroke (IS) and may worsen IS prognosis. Adverse effects of in-hospital infections on stroke outcome were also reported. We aimed to study the prevalence of pre- and poststroke infections and their impact on IS outcome. *Methods*. We analysed clinical data of 2066 IS patients to assess the effect of pre-stroke and post-stroke infections on IS severity, as well as short-term (up to 30 days) and long-term (90 days) outcome. The independent impact of infections on poor outcome (death, death/dependency) was investigated by use of logistic regression analysis. The effect of antibiotic therapy during hospitalization on the outcome was also assessed. *Results*. Pre-stroke infections independently predicted worse short-term outcome. In-hospital infections were associated with worse short-term and long-term IS prognosis. Antibacterial treatment during hospitalization did not improve patients' outcome. *Conclusions*. Prevention of infections may improve IS prognosis. The role of antibiotic therapy after IS requires further investigations.

## 1. Introduction

Infections preceding the ischemic stroke (IS), as well as infections occurring in the acute phase of IS, are a frequent phenomenon [1, 2]. Chronic infections of both viral and bacterial etiology and coexistent vascular inflammatory state promote atherosclerosis, contributing to an increased cerebrovascular risk [3, 4]. The association of prestroke acute infectious events, in particular, respiratory tract infections, with increased risk of stroke [5], especially of cardioembolic and atherothrombotic etiology, was reported [2, 6]. Some authors suggest that prestroke infections are related not only to the risk but also to IS severity [7, 8]. On the other hand, stroke severity and lesion location are associated with the risk of in-hospital (post-stroke) infections. For example, patients with brainstem or diffuse cerebral lesions characterize with an increased risk of respiratory tract infection, that frequently results from dysphagia [9, 10]. It seems possible that post-stroke infectious events are favoured by the stroke-induced immunodepression [2].

The impact of post-stroke infections on IS outcome is the next important issue [11]. Some authors describe an association of post-stroke infections with poor patients' outcome [12–14].

According to the presented data, a proper treatment of stroke-related infections may improve patients' outcome. Until now some reports suggest that therapy with antibiotics in the acute phase of stroke (even in patients without the obvious signs of infection) reduces cerebral ischemia and improves the outcome [15, 16]. However, these observations were not confirmed by other studies [17, 18]. Some authors hypothesized that the use of antibiotics or vaccines before stroke onset may improve outcome [19]. 

The aims of our study were to investigate the prevalence of prestroke (occurring during 7 days before hospital admission) and post-stroke (in-hospital) infections among IS patients hospitalized in our stroke unit, as well as to analyse the impact of infections on stroke severity and the outcome, in terms of mortality and dependency during 3 months after IS.

## 2. Methods

We analyzed the clinical data of patients hospitalized in our department between June 1, 1995 and August 31, 2005. The registry included 2066 consecutive patients admitted to the hospital with symptoms of acute IS. IS was diagnosed according to the World Health Organization definition as rapidly developed clinical signs of cerebral function disturbance lasting for more than 24 hours, of a vascular origin [20]. Brain imaging (CT, computed tomography or MRI, magnetic resonance imaging) was performed to confirm the diagnosis of IS. Information about stroke risk factors and clinical data was collected according to the modified Stroke Data Bank NINCDS protocol [21] including data on: age and gender, history of hypertension, diabetes, atrial fibrillation, coronary artery disease (CAD), heart failure and stroke, nicotine use during last 5 years, premorbid handicap status (dependency), neurological status on admission, etiology of IS, clinical stroke syndromes, volume of ischemic lesion, as well as infectious events during 7 days before admission, occurrence of pneumonia, and urinary tract infection (UTI) during hospitalization and the use of antibiotics during hospitalization. 

Baseline neurological status and premorbid functional impairment were evaluated using the Scandinavian Stroke Scale (SSS) (score 0–58) [22] and modified Rankin Scale (mRS) (score 0–5) [23].

Information about prestroke factors was obtained during direct interview with patients. In case of unconscious, drowsy, or demented patients, their close relatives were interviewed. The information was supported by medical reports, if available. The history of prestroke infection included reported signs of an acute infectious disease (such as cold, fever, cough, or dysuria), medical reports on such a condition, as well as the use of antibacterial, antiviral, or supportive therapy and laboratory findings obtained during preceding 7 days that were typical for an acute infection. In our database information on the type of prestroke infection, as well as on classes of antibiotics used prior to IS, is not collected, thus, we did not analyse such data. 

The etiology of IS was determined according to the criteria developed for the Trial of Org 10172 in Acute Stroke Treatment [24] on the basis of results of extra- and intracranial arteries ultrasonography, echocardiography, and electrocardiography. The five categories of IS were isolated according to etiology, that is, (1) large artery atherosclerosis, (2) cardioembolism, (3) small artery occlusion, (4) other determined etiology, and (5) unknown or multiple etiologies. In addition, using clinical criteria of the Oxfordshire Community Stroke Project (OCSP) based on neurological signs and syndromes, subtypes of IS were classified into total anterior circulation infarction (TACI), partial anterior circulation infarction (PACI), lacunar (LACI) and posterior circulation infarction (POCI) [25]. 

The diagnosis of pneumonia during hospitalization was established according to the CDC criteria for hospital-acquired pneumonia [26], on a basis of clinical and laboratory indices of respiratory tract infection (fever, cough, auscultatory changes, purulent tracheal secretions, positive sputum culture), supported by typical chest X-ray findings. The in-hospital UTI was diagnosed on a basis of leukocyturia present in the urine examination and positive urine culture (significant bacteriuria ≥ 10^5^/mL with isolation of a pathogen), supported by clinical symptoms, such as dysuria, urgency, or fever [26]. 

The SSS and mRS assessments were repeated at discharge or (if patient needed longer hospitalization) at the 30th day of hospital stay. The mRS score was also assessed 90 days after stroke. 

The short-term (assessed at discharge or at the 30th day of hospital stay) and long-term (assessed 90 days after IS onset) outcomes were analysed. The outcome measurements included (1) mortality; (2) death or dependency (mRS ≥ 3). Poor outcome was defined as death or death/dependency. In case of death within 90 days from the hospital admission, the date and reason were registered. The following causes of death were registered in the studied group: neurological complications (IS or recurrent stroke), cardiogenic reasons or pulmonary embolus, infectious complications, and others, not related to stroke.

### 2.1. Statistical Analysis

The groups of patients with or without the history of prestroke infection were compared in terms of prevalence of IS risk factors, premorbid handicap level (mRS), baseline clinical characteristics (IS etiology according to the TOAST criteria, clinical syndromes determined by the OCSP criteria, neurological status determined by the SSS score, body temperature recorded during first 24 hours, and 7 days mean body temperature). The groups of patients were also compared for stroke recurrence during hospitalization, for 30-day neurological status, as well as for short-term and long-term outcome. Similar comparisons was made between the groups of patients, in whom pneumonia or UTI were diagnosed after IS, and without any concomitant (prestroke) infection. 

The impact of prestroke and post-stroke infections on the short-term and long-term outcome (in terms of death and death/dependency) was investigated. 

Categorical variables were compared by the use of Pearson *χ* ^2^ test. The normality of continuous variables was determined by the Kolmogorow-Smirnov test. Normally distributed continuous variables were compared using the Student's *t*-test, and continuous variables with not normal distribution were compared with the Mann-Whitney *U* test. 

The mRS and SSS were evaluated by the *χ* ^2^ test, after dichotomizing the scales into categories: 0–2 and 3–5 points for the mRS, 0–25 and 26–58 points for the SSS, as it was accepted in previous studies [27, 28]. 

For the outcome measures (short- and long-term death and death/dependency) we did the Pearson *χ* ^2^ tests to evaluate differences between analysed groups. The logistic regression was used to assess a predictive value of prestroke and post-stroke infections for the outcome. Logistic regression analyses were performed, with adjustment for baseline and clinical variables. Variables that were significantly associated with a given outcome in univariate analysis were further included in the multivariate models. The odds ratios with 95% confidence intervals were used to estimate the magnitude of each factor effect. 

Results were thought statistically significant when the *P* value was lower than  .05. Statistical analyses were performed using SPSS for Windows software, version 13.

## 3. Results

Among 1950 patients for whom the necessary data was available, 111 (5.7%) had a history of an acute infection during 7 days before hospital admission.

Patients who suffered from an infectious disease prior to IS onset were characterized by older age, higher prevalence of heart failure, as well as by worse neurological status on hospital admission (Table 1). The prestroke infection was not related to the type of IS according to the TOAST criteria, nor to the volume of brain ischemic lesion. Patients with infection preceding stroke onset were characterized by poorer short- and long-term outcome in terms of death as well as of death/dependency, compared with those without infection (Table 1). 

30.6% versus 15.3% of patients with versus without prestroke infection, respectively, died during 30 days after stroke onset; the percentage of patients with/without prestroke infection who died within 90 days was 41.1%/25.2%, respectively. The poor short-term outcome in terms of death/dependency was noticed in 68.5% of patients with and in 52.9% of patients without prestroke infection; the poor long-term outcome was observed in 61.4% of patients with infection and in 49.1% of patients without prestroke infection (Table 1).

Among individuals with prestroke infection who died during the first 30 days the cardiogenic cause of death was diagnosed more frequently than in patients without infection (36.7% and 19.1%, resp.; *P* = .033). 

Logistic regression analysis revealed that the history of prestroke infection was an independent predictor of poor short-term outcome in terms of death and of death/dependency (Table 3); the prestroke infection was not a significant predictor of long-term outcome. 

We observed a higher frequency of in-hospital infections (pneumonia or UTI) in patients, who suffered from any infection during 7 days prior to hospital admission, compared to the group without prestroke infection (58.6% versus 33.7%, resp.); *P* < .001. About 81% of patients with any prestroke infection developed in-hospital pneumonia.

During hospitalization, nosocomial pneumonia was diagnosed in 403 (19.5% of 2066) patients and UTI in 482 (23.3%) patients; in 154 (7.5%) patients both infections were diagnosed.

368 (91.3%) patients with pneumonia, 441 (91.5%) patients with UTI, and 133 (86.4%) with both conditions, all with a negative history of prestroke infection, were further analysed.

Patients with in-hospital infection (pneumonia or UTI, *n* = 619) were characterized by older age, higher percentage of females, more frequent history of stroke, atrial fibrillation, heart failure, CAD and diabetes, than patients without diagnosed infection (Table 2). The post-stroke pneumonia or UTI developed in patients that were characterized by worse premorbid functional status and by worse neurological status on admission. The in-hospital infections occurred more frequently in patients presenting with TACI and with ischemic lesions occupying at least one lobe of the brain (Table 2).

In-hospital pneumonia more frequently developed among patients with dysphagia (92; 86.8% of 106 patients), than in those without swallowing difficulties (267; 48.2% of 554 patients); (*P* < .001).

In patients with in-hospital infection (pneumonia or UTI) the cardioembolic etiology of stroke was diagnosed more often; lacunar strokes were diagnosed less frequently, compared to patients without an infection (Table 2). 

The in-hospital infection was associated with a worse neurological status assessed at hospital discharge/30 days after admission; it was also related to worse short-term and long-term outcome in terms of death and of death/dependency (Table 2). 

We observed no influence of in-hospital infections on the prevalence of recurrent stroke during the hospitalization.

The regression analysis was performed to assess the impact of pneumonia, UTI, or of both coexisting conditions, on patients' outcome (Tables 4 and 5). The in-hospital pneumonia was independently associated with the risk of poor short-term and long-term outcome. The in-hospital UTI was independently associated with poor short- and long-term outcome in terms of death/dependency; however it was surprisingly associated with lower short-term mortality. Patients with coexistent pneumonia and UTI had an increased risk of poor short- and long-term outcomes in terms of death and of death/dependency (Tables 4 and 5). 

The mean body temperature recorded during the first 24 hours after admission was significantly higher in the group of patients suffering from an acute infection in the preceding week (Table 1). The body temperature at the first day after admission was significantly higher in patients with TACI, than in patients with other clinical syndromes (36.8°C ± 0.6 versus 36.7°C ± 0.4, resp.; *P* < .001); the same was noticed when the mean body temperature in the first 7 days after hospital admission was compared between patients' groups with TACI or with other clinical syndromes (37.1°C ± 0.4 versus 36.8°C ± 0.3, resp.; *P* < .001). 

In the regression analysis, increased temperature at the first day of hospital stay was a predictor of increased risk of death during 30 days in the group of patients with pneumonia and UTI coexisting; it was also a predictor of higher risk of short-term death/dependency in all groups of patients analysed (with pneumonia, UTI or with both infections) (Table 4). 

The increased mean week body temperature was an independent predictor of poor short-term and long-term outcomes in all groups of patients analysed (Tables 4 and 5).

In 365 (91.0%) patients with in-hospital pneumonia antibiotics were used during hospitalization. In this group of patients, antibacterial therapy had no significant impact on prognosis during the follow-up period. Among patients with UTI, 391 (81.8%) were treated with antibiotics. In this group, antibacterial therapy was related to poor 30-day outcome (in terms of death/dependency) that was noticed in 75.5% of the treated compared to 61.2% of not treated patients (*P* = .029).

Among 343 patients (16.6% of 2066), who died within the 30-day period, 83 (28.4%) died due to infective complications such as pneumonia or sepsis. The other causes of death were neurological, cardiogenic or unrelated to stroke. The presence of in-hospital pneumonia, or UTI was not related to death etiology. Patients with coexisting pneumonia and UTI more frequently died because of infectious complications, and less often because of neurological complications, than those without these infections. The treatment with antibiotics did not determine the cause of death in patients with pneumonia, with UTI, as well as with both conditions.

## 4. Discussion

We provide evidence that prestroke and post-stroke infections are independent predictors of poor IS outcome. This finding is consistent with other reports, which demonstrated the unfavourable impact of nosocomial infections, especially pneumonia, on short-term [13] and long-term stroke outcome [14, 29]. In our study the prestroke infection had an adverse effect on early outcome, with no effect on long-term prognosis, while the unfavourable effect of in-hospital infections (especially of pneumonia and UTI coexisting) was observed up to 90 days after the IS.

Some authors revealed that an association exists between prestroke respiratory infections and atherothrombotic and cardioembolic etiology of IS, as well as with more severe neurological deficit [30]. In the current study we confirmed an unfavourable effect of prestroke infections on neurological impairment; however, we did not confirm a relationship between prestroke infection and IS etiology.

The association of infarct volume with in-hospital infections, particularly the respiratory tract infection, has been previously described [31]. In the current study we have observed a similar relationship: patients with in-hospital pneumonia or UTI were characterized by more diffuse ischemic lesions (occupying at least one lobe or TACI). The relationship between lesion location and respiratory tract infection was also noticed: pneumonia occured more frequently in patients with dysphagia caused by brainstem or diffuse hemispheric stroke, as it was observed by previous authors [9, 10].

It was evidenced that high body temperature during first 24 hours after stroke onset is associated with large brain damage [32]. In our population increased body temperature during the first 7 days of hospital stay was associated with TACI and diffuse ischemic lesions. The increased body temperature during the first week of hospitalization was independently related to worse short-term and long-term outcome, even after adjustment for in-hospital infection. These findings lead to the conclusion that increased body temperature during the acute phase of IS should be reduced as soon as possible, in order to improve patients' prognosis. 

It seems interesting that, in the analysed group of IS patients, the highest risk of developing pneumonia or UTI during hospitalization was noticed in older females as well as in patients with coexisting cardiovascular risk factors like atrial fibrillation, heart failure, CAD, and diabetes, as well as in those with a history of prestroke infection. It suggests that such patients require a particularly careful prevention and early detection of infectious diseases. 

When analysing the antibacterial treatment in the studied population, our results are not optimistic. The therapy with antibiotics during hospitalization in patients with pneumonia had no significant impact on the outcome. In the group of patients with UTI, an unfavourable effect of antibacterial treatment on short-term outcome (death/dependency) was observed. It seems surprising; however, it may be probably partially explained by the fact that UTI occurs often in patients catheterised, so in a worse global condition. The lack of favourable effect of antibacterial therapy on IS outcome indicates that prophylaxis of nosocomial pneumonia or UTI is important, as it may improve patients' outcome. 

We acknowledge that our study has several limitations. First, we did not analyse the pre- and post-stroke antibacterial therapy in detail (including the type of antibiotics, dose, and duration of treatment, as well as the time of implementation of antibiotics after IS onset). When considering post-stroke antibacterial treatment, typically, in our department, in patients with symptoms of pneumonia the antibiotics of wide therapeutical spectrum are used and then, after detection of the pathogen, specific classes of antibiotics are implemented. However, it could not be excluded that some patients might have pneumonia of the viral etiology, but because of their poor condition they were also treated with antibiotics. It might potentially influence our findings concerning the effects of antibacterial therapy. 

Furthermore, when analyzing the impact of prestroke infections on the outcome, we focused on acute infection symptoms present during the 7 days before stroke onset. It is obvious that infections potentially related to stroke can occur up to several weeks prior to stroke onset; however, the major effect on stroke risk seems to have infections occuring during the preceding week [33].

## 5. Conclusions

Pre- and post-stroke infections, particularly those occurring during hospitalization, are associated with a worse IS prognosis. The increase of body temperature during first 7 days after IS, even in patients without detected infection, has an adverse impact on their short-term and long-term outcome. The treatment with antibiotics during hospitalization in IS patients has no evident beneficial effect; this issue should be further investigated. The importance of prevention of pneumonia and UTI in patients with IS should be emphasized.

##  Conflict of Interests

The authors declare no conflict of interests. 

## Figures and Tables

**Table 1:** Characteristics of patients with or without prestroke infection.

Characteristics	Prestroke infection (*n* = 111)		No prestroke infection (*n* = 1839)		*P*
*N*	Value	*N*	Value

*Baseline characteristics*					
Age, mean, SD (years)	111	74.6 ± 11.8	1834	71.5 ± 12.3	.010
Gender (male), *n* (%)	111	49 (44.1)	1839	843 (45.8)	.769
Premorbid mRS 3–5, *n* (%)	111	17 (15.3)	1833	178 (9.7)	.071
History of stroke, *n* (%)	110	18 (16.4)	1818	283 (15.6)	.788
Hypertension, *n* (%)	109	74 (67.9)	1830	1353 (73.9)	.179
Atrial fibrillation, *n* (%)	110	30 (27.3)	1824	513 (28.1)	.913
Heart failure, *n* (%)	109	37 (33.9)	1814	417 (23.0)	.014
CAD, *n* (%)	110	43 (39.1)	1825	646 (35.4)	.473
Diabetes, *n* (%)	111	25 (22.5)	1823	397 (21.8)	.814
Nicotine use (5 years), *n* (%)	107	21 (19.6)	1822	476 (26.1)	.141
Etiology of IS (TOAST)	111		1839		
Atherothrombotic, *n* (%)		16 (14.4)		291 (15.8)	.789
Cardioembolic, *n* (%)		28 (25.2)		484 (26.3)	.912
Lacunar, *n* (%)		16 (14.4)		343 (18.7)	.313
Diffuse lesion (≥1 lobe)	82	33 (40.2)	1110	384 (34.6)	.337
TACI, *n* (%)	110	26 (23.6)	1830	306 (16.7)	.068
Admission SSS, mean, SD	111	29.2 ± 18.0	1836	34.0 ± 17.3	.008
Body temperature day 1, mean (°C)	100	36.8 ± 0.58	1724	36.7 ± 0.43	.033
Mean week body temperature (°C)	80	36.9 ± 0.34	1432	36.8 ± 0.36	.038

*Outcome*					
Recurrent stroke, *n* (%)	111	1 (0.9)	1837	29 (1.6)	.573
30-day SSS, mean, SD	80	43.9 ± 13.9	1564	46.1 ± 13.4	.157
30-day mortality, *n* (%)	111	34 (30.6)	1839	281 (15.3)	<.001
30-day death or dependency, *n* (%)	111	76 (68.5)	1809	957 (52.9)	.002
90-day mortality, *n* (%)	95	39 (41.1)	1601	404 (25.2)	.001
90 day death or dependency, *n* (%)	88	54 (61.4)	1514	743 (49.1)	.028

*N*: the number of patients with available data; SD: standard deviation; mRS: modified Rankin Scale; CAD: coronary artery disease; TACI: total anterior circulation infarction; IS: ischemic stroke; TOAST: Trial of Org 10172 in Acute Stroke Treatment; SSS: Scandinavian Stroke Scale.

**Table 2:** Characteristics of patients with or without an in-hospital infection.

Characteristics	Pneumonia or UTI (*n* = 619)		No infection (*n* = 1219)		*P*
*N*	Value	*N*	Value

*Baseline characteristics*					
Age, mean, SD (years)	618	76.1 ± 10.6	1215	69.2 ± 12.4	<.001
Gender (male), *n* (%)	619	201 (32.5)	1219	641 (52.6)	<.001
Premorbid mRS 3–5, *n* (%)	616	95 (15.4)	1216	83 (6.8)	<.001
History of stroke, *n* (%)	614	114 (18.6)	1203	169 (14.0)	.014
Hypertension, *n* (%)	614	456 (74.3)	1215	896 (73.7)	.822
Atrial fibrillation, *n* (%)	613	234 (38.2)	1210	279 (23.1)	<.001
Heart failure, *n* (%)	604	193 (32.0)	1209	223 (18.4)	<.001
CAD, *n* (%)	613	247 (40.3)	1211	398 (32.9)	.002
Diabetes, *n* (%)	613	158 (25.8)	1209	239 (19.8)	.004
Nicotine use (5 years), *n* (%)	615	125 (20.3)	1206	351 (29.1)	<.001
Etiology of IS (TOAST)	616		1213		
Atherothrombotic, *n* (%)		93 (15.1)		114 (9.4)	.543
Cardioembolic, *n* (%)		209 (33.9)		275 (22.7)	<.001
Lacunar, *n* (%)		61 (9.9)		282 (23.2)	<.001
Diffuse lesion (≥1 lobe)	408	211 (51.7)	701	173 (24.7)	<.001
TACI, *n* (%)	616	192 (31.2)	1213	114 (9.4)	<.001
Admission SSS, mean, SD	617	23.4 ± 16.1	1218	39.3 ± 15.4	<.001
Body temperature day 1, mean (°C)	574	36.8 ± 0.52	1149	36.7 ± 0.37	<.001
Mean week body temperature (°C)	616	36.9 ± 1.27	1208	36.7 ± 0.79	<.001

*Outcome*					
Recurrent stroke, *n* (%)	619	14 (2.3)	1219	15 (1.2)	.113
30-day SSS, mean, SD	424	36.7 ± 15.9	1139	49.6 ± 10.3	<.001
30-day mortality, *n* (%)	619	192 (31.0)	1219	89 (7.3)	<.001
30-day death or dependency, *n* (%)	599	504 (84.1)	1209	453 (37.5)	<.001
90-day mortality, *n* (%)	534	274 (51.3)	1066	130 (12.2)	<.001
90-day death or dependency, *n* (%)	502	405 (80.7)	1011	338 (33.4)	<.001

UTI: urinary tract infection; *N*: the number of patients with available data; SD: standard deviation; mRS: modified Rankin Scale; CAD: coronary artery disease; TACI: total anterior circulation infarction; IS: ischemic stroke; TOAST: Trial of Org 10172 in Acute Stroke Treatment; SSS: Scandinavian Stroke Scale.

**Table 3:** Logistic regression for poor 30-days outcome (prestroke infection).

	Variables	OR	95% CI	*P*
Death	*Prestroke infection*	2.086	1.323–3.288	.002
	Age	1.036	1.023–1.050	<.001
	Gender (male)	0.934	0.709–1.230	.627
	mRS 3–5 before stroke	1.191	1.078–1.317	.001
	Heart failure	2.038	1.516–2.740	<.001
	Atrial fibrillation	1.413	1.065–1.876	.017
	Diabetes mellitus	1.296	0.963–1.745	.087
	CAD	0.912	0.688–1.208	.519

Death or dependency	*Prestroke infection*	1.635	1.044–2.560	.032
	Age	1.027	1.018–1.037	<.001
	Gender (male)	0.807	0.657–0.992	.041
	mRS 3–5 before stroke	1.963	1.728–2.230	<.001
	History of stroke	0.904	0.666–1.227	.519
	Heart failure	1.198	0.916–1.566	.187
	Atrial fibrillation	1.345	1.053–1.716	.017
	Diabetes mellitus	1.231	0.962–1.574	.098
	CAD	1.081	0.865–1.351	.492

OR: odds ratio; CI: confidence interval; mRS: modified Rankin Scale; CAD: coronary artery disease.

**Table 4:** Logistic regression for poor 30-day outcome (in-hospital infections).

	Variables	Pneumonia			UTI			Pneumonia and UTI		
		OR	95% CI	*P*	OR	95% CI	*P*	OR	95% CI	*P*

Death	*Pneumonia*	5.422	3.684–7.979	<.001	—	—	—	—	—	—
	*UTI*	—	—	—	0.551	0.353–0.859	.009	—	—	—
	*Pneumonia and UTI*	—	—	—	—	—	—	2.275	1.454–3.559	<.001
	Age	1.028	1.012–1.044	<.001	1.038	1.022–1.054	<.001	1.037	1.021–1.052	<.001
	Gender (male)	0.850	0.605–1.194	.350	0.912	0.656–1.266	.581	0.987	0.711–1.371	.939
	mRS 3–5 before stroke	1.699	1.093–2.641	.018	1.652	1.073–2.543	.023	1.598	1.043–2.450	.031
	Heart failure	2.197	1.527–3.162	<.001	2.079	1.463–2.954	<.001	2.018	1.419–2.870	<.001
	Atrial fibrillation	1.143	0.806–1.623	.453	1.206	0.861–1.688	.276	1.193	0.850–1.673	.307
	Diabetes mellitus	1.093	0.757–1.576	.636	1.108	0.775–1.584	.574	1.108	0.776–1.583	.574
	CAD	0.865	0.611–1.225	.415	0.875	0.625–1.224	.435	0.856	0.612–1.198	.365
	Body temperature day 1	1.413	0.968–2.061	.073	1.384	0.958–2.000	.083	1.467	1.017–2.117	.041
	Mean week temperature	3.528	2.376–5.238	<.001	4.906	3.334–7.219	<.001	3.963	2.663–5.898	<.001

Death or dependency	*Pneumonia*	12.467	6.449–24.102	<.001	—	—	—	—	—	—
	*UTI*	—	—	—	2.125	1.536–2.942	<.001	—	—	—
	*Pneumonia and UTI*	—	—	—	—	—	—	20.615	6.385–66.562	<.001
	Age	1.027	1.017–1.038	<.001	1.032	1.022–1.042	<.001	1.032	1.022–1.042	<.001
	Gender (male)	0.700	0.556–0.882	.002	0.847	0.674–1.063	.152	0.783	0.624–0.981	.034
	mRS 3-5 before stroke	17.244	8.495–35.004	<.001	16.536	7.998–34.187	<.001	16.854	8.295–34.241	<.001
	Heart failure	1.301	0.960–1.763	.090	1.282	0.952–1.727	.102	1.252	0.926–1.693	.145
	Atrial fibrillation	1.152	0.875–1.515	.313	1.185	0.907–1.548	.213	1.138	0.868–1.492	.348
	Diabetes mellitus	1.288	0.978–1.696	.071	1.265	0.966–1.657	.088	1.309	0.997–1.719	.052
	CAD	1.147	0.894–1.472	.281	1.104	0.864–1.412	.428	1.142	0.891–1.462	.294
	Body temperature day 1	1.963	1.391–2.772	<.001	1.895	1.348–2.665	<.001	1.937	1.380–2.719	<.001
	Mean week temperature	2.262	1.648–3.105	<.001	2.946	2.089–4.155	<.001	2.288	1.636–3.200	<.001

OR: odds ratio; CI: confidence interval; mRS: modified Rankin Scale; CAD: coronary artery disease.

**Table 5:** Logistic regression for poor 90-day outcome (in-hospital infections).

	Variables	Pneumonia			UTI			Pneumonia or UTI		
		OR	95% CI	*P*	OR	95% CI	*P*	OR	95% CI	*P*

Death	*Pneumonia*	4.345	2.876–6.564	<.001						
	*UTI*				0.756	0.513–1.116	.159			
	*Pneumonia and UTI*							5.730	3.399–9.660	<.001
	Age	1.043	1.028–1.058	<.001	1.049	1.034–1.064	<.001	1.048	1.033–1.063	<.001
	Gender (male)	0.973	0.716–1.322	.861	1.040	0.770–1.404	.800	1.089	0.804–1.477	.582
	mRS 3–5 before stroke	2.122	1.365–3.299	.001	2.055	1.328–3.180	.001	2.063	1.328–3.204	.001
	Heart failure	2.121	1.503–2.993	<.001	2.036	1.452–2.853	<.001	1.928	1.362–2.730	<.001
	Atrial fibrillation	1.357	0.984–1.870	.062	1.406	1.029–1.921	.032	1.367	0.992–1.884	.056
	Diabetes mellitus	1.030	0.735–1.443	.865	1.042	0.748–1.451	.808	1.029	0.733–1.443	.870
	CAD	0.944	0.687–1.297	.724	0.942	0.690–1.286	.706	0.955	0.694–1.315	.778
	Body temperature day 1	1.128	0.770–1.654	.536	1.076	0.738–1.568	.704	1.175	0.799–1.729	.413
	Mean week temperature	7.338	4.801–11.216	<.001	10.079	6.595–15.401	<.001	6.766	4.367–10.485	<.001

Death or dependency	*Pneumonia*	8.700	4.743–15.956	<.001						
	*UTI*				1.517	1.066–2.158	.020			
	*Pneumonia and UTI*							8.940	3.975–20.108	<.001
	Age	1.035	1.023–1.047	<.001	1.039	1.028–1.051	<.001	1.039	1.027–1.051	<.001
	Gender (male)	0.769	0.596–0.992	.043	0.902	0.702–1.159	.419	0.851	0.663–1.092	.205
	mRS 3–5 before stroke	8.850	4.648–16.852	<.001	8.206	4.287–15.707	<.001	8.515	4.468–16.228	<.001
	Heart failure	1.309	0.941–1.820	.110	1.284	0.930–1.774	.129	1.206	0.868–1.677	.264
	Atrial fibrillation	1.259	0.937–1.692	.127	1.275	0.956–1.700	.099	1.241	0.926–1.664	.148
	Diabetes mellitus	1.350	1.005–1.813	.046	1.344	1.007–1.794	.045	1.362	1.016–1.826	.039
	CAD	1.235	0.939–1.626	.131	1.205	0.920–1.576	.175	1.252	0.954–1.644	.105
	Body temperature day 1	1.226	0.838–1.794	.295	1.207	0.834–1.748	.318	1.306	0.898–1.899	.162
	Mean week temperature	3.250	2.151–4.908	<.001	4.521	2.969–6.886	<.001	3.238	2.114–4.962	<.001

OR: odds ratio; CI: confidence interval; mRS: modified Rankin Scale; CAD: coronary artery disease.
